# Valorization of waste oyster shells *via* thermal and acid activation for Congo red dye adsorption from aqueous media

**DOI:** 10.1039/d5ra08441e

**Published:** 2026-01-02

**Authors:** Huynh Nhi Le, Hoai Phuong Nguyen Thi, Phuong Anh Cao, Ba Cuong Nguyen, Van Bang Nguyen, Duong Duc La

**Affiliations:** a Le Quy Don University 236 Hoang Quoc Viet Hanoi Vietnam; b Joint Vietnam-Russia Tropical Science and Technology Research Center 63 Nguyen Van Huyen Hanoi Vietnam; c Institute of Materials, Biology, and Environment 17 Hoang Sam Hanoi Vietnam duc.duong.la@gmail.com

## Abstract

A scalable route to valorize waste oyster shells into an effective adsorbent for Congo red removal is reported. Sequential thermal calcination (500 °C) and H_3_PO_4_ activation convert the CaCO_3_ matrix into Ca–phosphate-rich surfaces (XRD, FTIR) bearing abundant –OH/PO_4_ groups. Despite a moderate BET area (MOS: 22.15 m^2^ g^−1^), the modified oyster shell achieves rapid uptake (>80–90% removal within 10 min; near-complete by 60 min), broad pH tolerance with optimal performance below pH_pzc_ ≈ 8.06, and high capacity (*q*_max_ = 50.89 mg g^−1^). Kinetics follow a pseudo-second-order model (*R*^2^ = 0.994; *k*_2_ = 0.0127 g mg^−1^ min^−1^) and equilibrium data fit both Freundlich (*R*^2^ = 0.997; *K*_F_ = 37.19; *n* = 2.97) and Langmuir (*K*_L_ = 4.21 L mg^−1^) models, indicating chemisorptive affinity on an energetically heterogeneous surface. MOS is durable and regenerable (∼95% removal after five cycles). Density functional theory calculations corroborate strong dye-phosphate site interactions. The combined thermal-acid treatment thus yields a low-cost, reusable adsorbent suitable for practical dye-laden wastewater treatment.

## Abstract 2

A scalable route to valorize waste oyster shells into an effective adsorbent for Congo red removal is reported.

## Graphical abstract

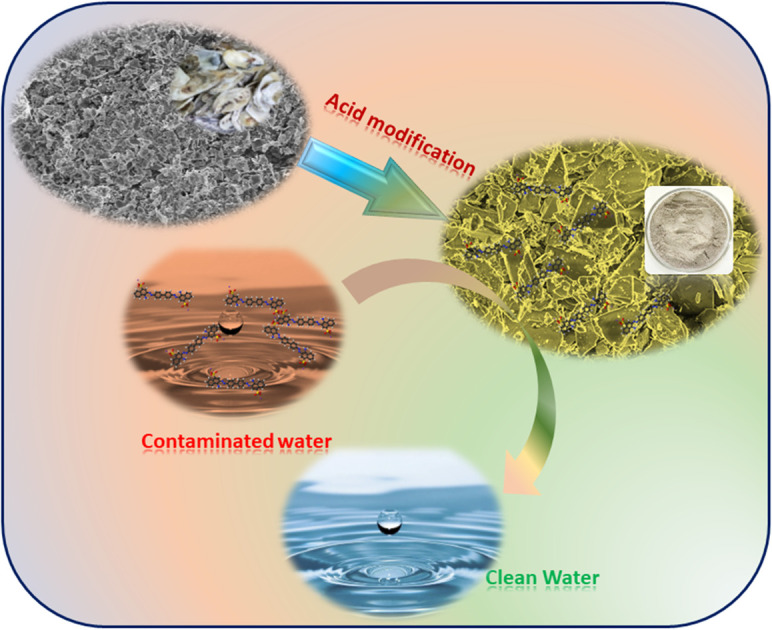

## Introduction

Synthetic dye pollution has emerged as a major environmental challenge associated with textile, printing, paper, and dyeing industries, causing serious ecological and health hazards worldwide.^1–3^ Dyes are often released in large quantities into water bodies, where they impede photosynthesis, disrupt aquatic ecosystems, and reduce water quality.^4,5^ Among these pollutants, Congo red (CR), an anionic benzidine-based azo dye, is particularly concerning due to its high solubility, strong color intensity, and resistance to biodegradation.^6–8^ Even at trace concentrations, CR imparts visible coloration to water, reduces light penetration, and can exert mutagenic and carcinogenic effects on living organisms.^7,8^ Owing to its structural complexity and stability, conventional wastewater treatments such as coagulation, oxidation, and biological degradation often fail to effectively remove CR.^9,10^ Therefore, developing cost-effective, sustainable, and efficient adsorbents remains a crucial route for mitigating dye-induced water contamination.

Among various treatment methods, adsorption stands out for its simplicity, high efficiency, and adaptability under diverse operational conditions.^11–13^ Activated carbon remains the benchmark adsorbent due to its large surface area and tunable porosity;^14,15^ however, its high production and regeneration cost limits scalability. To address this, recent studies have emphasized the valorization of bio-wastes and low-cost materials as eco-friendly adsorbents.^16–18^ Biosorbents derived from lignocellulosic waste, fruit peels, rice husks, eggshells, and marine shells demonstrate remarkable potential in dye and metal removal while supporting circular economy goals.^15,16^ In particular, seashell-derived materials, rich in calcium carbonate (CaCO_3_), have attracted attention for their natural alkalinity, ion-exchange capacity, and abundant availability.^17–19^ These materials can be thermally or chemically activated to enhance porosity, generate reactive surface sites, and improve affinity for anionic pollutants such as Congo red. Comparable properties have been reported in layered double hydroxides (LDHs), which exhibit tunable interlayer anion-exchange behavior and high surface basicity, making them effective for pharmaceutical and dye wastewater treatment.^20^

Oyster shells, a dominant by-product of aquaculture and seafood industries, represent a massive waste stream posing disposal and environmental concerns.^21–23^ Globally, millions of tons of oyster shells are discarded annually, often in landfills or coastal zones, where they contribute to odor, microbial growth, and pollution. Structurally, oyster shells consist primarily of CaCO_3_ (as calcite and aragonite) with trace organic matter, offering a suitable precursor for adsorbent synthesis.^24,25^ Through calcination, CaCO_3_ can be transformed into reactive CaO, increasing alkalinity and surface reactivity. Subsequent acid modification introduces functional phosphate groups, enhancing surface acidity and adsorption potential through electrostatic interaction, ion exchange, and hydrogen bonding.^26–28^ Similar phosphate-based modification has also improved the adsorption performance of Mg/Fe-LDH composites for heavy metal removal, confirming the beneficial role of phosphonate ligands in binding strength and surface heterogeneity.^29^

Previous research has shown that modified oyster shells effectively remove heavy metals and organic pollutants.^30–32^ Nevertheless, most studies focus on single-step activation or individual modification routes, leaving the combined effect of thermal activation and phosphoric acid modification largely unexplored. This knowledge gap is critical, as a synergistic activation strategy could simultaneously improve surface porosity, functional group density, and adsorption capacity. Moreover, while advanced photocatalytic hybrids such as RGO/g-C_3_N_4_–WO_3_/Bi_2_WO_6_ have achieved efficient organic degradation under visible light,^33^ their synthesis is often complex and costly, limiting large-scale application. In contrast, phosphoric-acid-modified oyster shell (MOS) offers a sustainable, low-cost, and scalable approach for dye adsorption in aqueous environments.

In this study, discarded oyster shells were valorized through a combined thermal–acid modification route to produce a phosphate-enriched biosorbent for efficient Congo red removal. The synergistic activation enhanced surface functionality and adsorption affinity, transforming a problematic waste into a high-value material. This work bridges waste valorization and water purification, offering a sustainable and economically viable solution for dye-contaminated wastewater.

## Experimental section

### Materials

Waste oyster shells (WOS) were obtained from seafood processing facilities in Quang Ninh Province, Vietnam. The unprocessed shells were meticulously rinsed with tap water to eliminate organic residues, boiled for one hour, and subsequently dried in an oven at 105 °C for 24 hours. The desiccated shells were subsequently pulverized and sifted to acquire particles smaller than 200 µm. Analytical grade phosphoric acid (H_3_PO_4_, 85%), Congo red dye (C_32_H_22_N_6_Na_2_O_6_S_2_, MW 696.66 g mol^−1^), sodium hydroxide (NaOH), and hydrochloric acid (HCl) were acquired from Sigma-Aldrich. All solutions were formulated utilizing deionized (DI) water.

### Preparation of modified oyster shell (MOS) adsorbent

Adsorbents produced from oyster shells were synthesized: Dried oyster shell powders were subjected to calcination in a muffle furnace at 500 °C for 2 hours, with a heating rate of 10 °C min^−1^, followed by cooling in a desiccator. The calcined material was treated with a 0.5 M H_3_PO_4_ solution at a solid-to-liquid ratio of 1 : 7.5 (w/v) while being continuously stirred at 65 °C for 2 hours. The slurry underwent filtration, was rinsed with deionized water until achieving a neutral pH, was oven-dried at 105 °C, and was subsequently crushed into a fine powder. The acquired materials were preserved in airtight containers and utilized as adsorbents in later tests.

### Characterization of adsorbents

The physicochemical characteristics of the unaltered and modified oyster shells were methodically examined. Scanning electron microscopy (SEM) was utilized to examine surface morphology. X-ray diffraction (XRD, Cu Kα radiation, *λ* = 1.5406 Å) was employed to ascertain crystalline phases. Functional groups were identified using Fourier-transform infrared spectroscopy (FTIR) in the range of 4000–400 cm^−1^. The Brunauer–Emmett–Teller (BET) surface area and pore volume were measured using nitrogen (N_2_) gas at the point of zero charge (pH_pzc_) was ascertained utilizing the pH drift method.

### Batch adsorption experiments

Adsorption experiments were conducted in 250 mL Erlenmeyer flasks containing 100 mL of Congo red solution at specified concentrations (10–200 mg L^−1^). A specified dosage of adsorbent (0.1–1.0 g L^−1^) was introduced, and the flasks were stirred at 150 rpm in a temperature-controlled shaker at the room temperatures (30 ± 2 °C). The pH of the solution (2–10) was modified utilizing 0.1 M HCl or 0.1 M NaOH. At designated time intervals, samples were extracted, centrifuged, and assessed for residual dye concentration utilizing a UV-Vis spectrophotometer at *λ*_max_ = 497 nm. The adsorption capacity (*q*_e_, mg g^−1^) and removal efficiency (*R*, %) were determined using:1
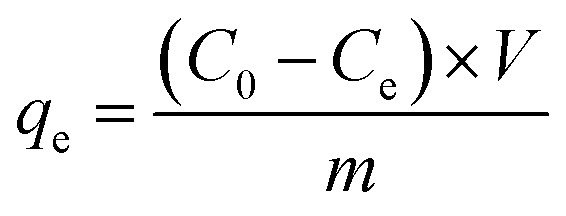
2
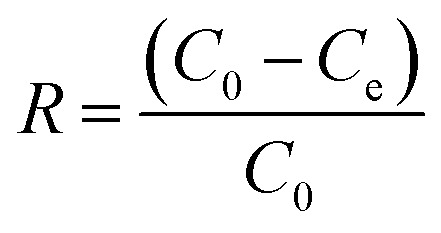
where *C*_0_ and *C*_e_ designate the starting and equilibrium dye concentrations measured in mg L^−1^, *V* signifies the solution volume in liters, and *m* represents the mass of the adsorbent in grams.

The adsorption kinetics were examined utilizing pseudo-first-order, pseudo-second-order, Elovich, and intraparticle diffusion models. Equilibrium data were examined utilizing Langmuir, Freundlich, Temkin, and Elovich models.

### Regeneration and reusability studies

To evaluate the reusability of the adsorbent, we conducted desorption studies by immersing the dye-saturated oyster shell in 96% ethanol. The regenerated adsorbent was rinsed with deionized water, dried at 105 °C, and employed for seven consecutive adsorption–desorption cycles under the same conditions. The retention of efficiency was analyzed over cycles to assess regeneration performance.

## Results and discussion

### Characterization of modified oyster shell

**Fig. 1:**
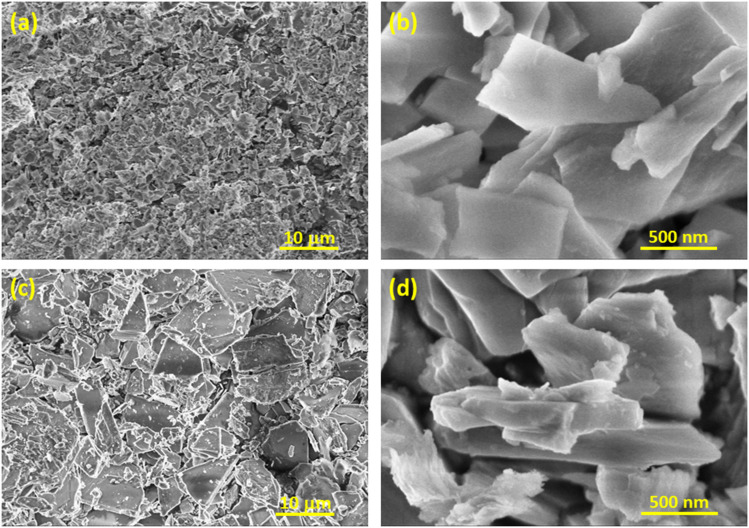
SEM images of waste oyster shells (a and b) and modified oyster shell (c and d) samples.

Fig. 1 presents SEM micrographs illustrating the morphological evolution of oyster shell surfaces before and after the combined thermal and phosphoric-acid modification.

The pristine oyster shell (Fig. 1a and b) exhibits a compact, plate-like morphology with tightly packed CaCO_3_ crystallites and smooth layered structures typical of aragonite and calcite phases. The absence of visible mesopores and the presence of dense, overlapping plates indicate limited external surface area and few accessible active sites for adsorption. Such morphology explains the relatively low adsorption capacity of the untreated material.

After calcination and subsequent phosphoric-acid activation (Fig. 1c and d), the surface undergoes a profound transformation. The modified sample displays a fragmented and highly corrugated texture, characterized by irregular cavities, fissures, and interparticle gaps. The edges of individual plates appear etched and roughened, reflecting partial dissolution of CaCO_3_ and formation of Ca–phosphate phases through acid–base reactions between CaO and H_3_PO_4_. These structural modifications markedly enhance surface heterogeneity and pore accessibility.

At higher magnification (Fig. 1d), loosely aggregated nanosheets and flake-like structures can be observed. Mild particle agglomeration is visible—likely resulting from local sintering during calcination—but the porous framework remains continuous and uniformly distributed. This morphology ensures reproducible adsorption performance and uniform dye accessibility throughout the surface. The coexistence of micro- and nano-scale roughness provides abundant active centers for electrostatic attraction, ion exchange, and hydrogen bonding with anionic dye molecules such as Congo red.

Overall, the SEM observations confirm that the synergistic thermal–acid activation converts the smooth CaCO_3_ matrix into a rough, porous Ca–phosphate composite with enhanced structural complexity. This hierarchical architecture underpins the superior adsorption efficiency of the modified oyster shell (MOS) adsorbent.

**Fig. 2:**
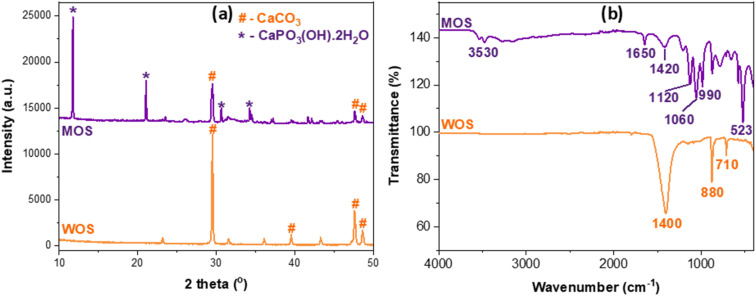
XRD pattern (a) and FTIR spectra (b) of waste oyster shells and modified oyster shell samples.

Fig. 2 presents the XRD and FTIR analyses of waste oyster shell (WOS) and modified oyster shell (MOS) samples, highlighting the structural transformation from carbonate to phosphate phases after combined thermal and phosphoric-acid activation.

In the XRD patterns (Fig. 2a), WOS displays distinct diffraction peaks at 2*θ* ≈ 29.5°, 39.5°, 47.6°, and 48.6°, corresponding to the characteristic reflections of calcite CaCO_3_ (PDF 00-005-0586). These sharp peaks indicate a well-crystallized carbonate matrix typical of biogenic calcium carbonate materials such as oyster or eggshell powders.^34–36^ Upon modification, the diffraction profile of MOS changes markedly, showing new reflections at approximately 11.8°, 21.0°, 31.2°, and 34.5°, assigned to brushite [CaHPO_4_·2H_2_O≡CaPO_3_(OH)·2H_2_O] (PDF 00-009-0077). The attenuation of calcite peaks and the emergence of phosphate-related signals confirm the partial conversion of CaCO_3_ into Ca–phosphate phases through acid–base reactions between thermally produced CaO and H_3_PO_4_.^35,37^ This phase transformation enhances structural disorder and generates chemically active phosphate moieties that improve surface reactivity and adsorption affinity.

The FTIR spectra (Fig. 2b) further corroborate the XRD results. For WOS, the absorption bands at ≈1400 cm^−1^ (*ν*_3_ CO_3_^2−^ asymmetric stretching) and ≈880 and 710 cm^−1^ (*ν*_2_ and *ν*_4_ modes) correspond to typical carbonate vibrations in CaCO_3_.^36^ In contrast, MOS exhibits a broad O–H stretching band near 3530 cm^−1^ and an H–O–H bending vibration at 1650 cm^−1^ from structural and adsorbed water. Strong phosphate absorptions appear at 1120, 1060, and 990 cm^−1^ (*ν*_3_ PO_4_^3−^ stretching) and 523 cm^−1^ (*ν*_4_ PO_4_^3−^ bending), indicating the formation of Ca–phosphate species. A weak residual band near 1420 cm^−1^ is typical of partial carbonate substitution within brushite-type Ca–phosphate structures.^35–37^

The complementary XRD and FTIR evidence unequivocally demonstrates the structural conversion of CaCO_3_ into a Ca–phosphate-enriched composite. This transformation introduces abundant surface –OH and PO_4_^3−^ functional groups, enhancing polarity, charge heterogeneity, and binding affinity toward anionic dyes such as Congo red, thus directly explaining the improved adsorption performance of the modified oyster shell.

**Fig. 3:**
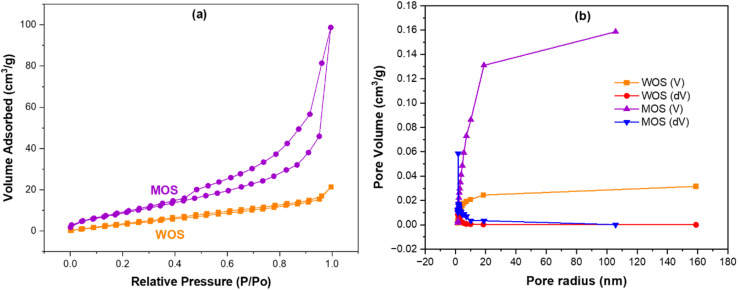
N_2_ adsorption isotherms (a) and pore-size distribution (b) of oyster shell samples.

As shown in Fig. 3a, both samples exhibit Type II isotherms with an H_3_ hysteresis loop according to IUPAC classification, characteristic of nonporous or macroporous solids with slit-like pores formed by plate-like particles. The gradual uptake at high relative pressure (*P*/*P*_0_ > 0.8) indicates capillary condensation within interparticle voids rather than within well-developed mesopores, consistent with carbonate-based materials.

Quantitative BET analysis revealed that WOS possesses a specific surface area (*S*_BET_) of 40.51 m^2^ g^−1^ and a total pore volume (*V*_tot_) of 0.159 cm^3^ g^−1^, while the MOS sample exhibits 22.15 m^2^ g^−1^ and 0.032 cm^3^ g^−1^, respectively. Despite the decrease after phosphoric-acid modification, these values are well within the typical range for shell-derived adsorbents (1–65 m^2^ g^−1^; 0.02–0.15 cm^3^ g^−1^).^38,39^ The decline in both surface area and pore volume for MOS can be attributed to phosphate precipitation and crystal growth during conversion of CaCO_3_ into Ca–phosphate phases, which partially fill or collapse interparticle mesopores, as also reflected in the SEM morphology.

The corresponding BJH pore-size distributions (Fig. 3b) show that WOS contains broader mesopores in the 10–50 nm range, while MOS exhibits a narrower distribution dominated by smaller mesopores and reduced macroporosity. Such evolution suggests partial pore blocking by deposited phosphate layers and restructuring of the surface into compact agglomerates, consistent with the phase transformation evidenced by XRD and FTIR analyses.

In addition, the molecular dimensions of Congo red (CR) are relatively large—approximately 2.6 nm in length and 0.7–1.0 nm in width—which is consistent with previous reports describing CR as an elongated azo dye with hindered diffusion in narrow pores, such as when used in MOF- or silk-nanofibril-based composite membranes.^40^ When the pore-size distribution of MOS is compared with the molecular size of CR, it becomes clear that the mesopores of MOS (predominantly >10 nm) are sufficiently larger than the dye molecule. Therefore, MOS does not impose a size-selective steric exclusion effect on CR. Instead, the adsorption process is governed primarily by surface interactions—protonated phosphate and hydroxyl groups enabling strong electrostatic attraction and ion exchange—rather than by molecular sieving or pore-diffusion constraints. This also explains the rapid uptake observed in kinetic studies, as CR is not physically hindered from accessing the external surface or mesoporous domains of MOS.

Although MOS displays lower textural parameters than WOS, the emergence of chemically active phosphate and hydroxyl groups significantly enhances its surface reactivity and adsorption affinity. This finding underscores that adsorption efficiency is governed not only by surface area but also by surface chemistry and functionality, explaining the improved dye uptake observed for MOS despite its reduced BET value.

Overall, the isotherm type, pore-size characteristics, and quantitative BET/BJH data are consistent with previous reports on CaCO_3_-based shells and their Ca–phosphate derivatives, confirming the successful modification and realistic textural features of the biosorbent.

### Effect of contact time and kinetics

**Fig. 4:**
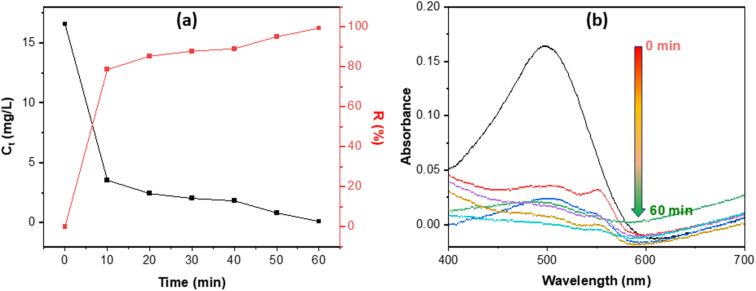
Congo red removal efficiency of MOS (a) and UV-Vis spectra (b) *vs.* contact time.

Fig. 4 illustrates that MOS effectively eliminates Congo red in a rapid manner: the dye concentration (*C*_t_) decreases sharply within the initial ∼10 minutes, achieving over 80% elimination, and subsequently nears total decolorization by 60 minutes as the curve stabilizes—indicative of rapid surface adsorption followed by gradual site saturation. The UV-Vis spectra (b) confirm this trend: the characteristic CR band at 497 nm gradually decreases and is nearly eliminated by 60 minutes, indicating effective dye removal from the solution. This behavior aligns with previous studies on seashell-derived and calcium-phosphate sorbents, where CR is consistently measured at *λ*_max_ ≈ 497 nm, exhibiting a rapid initial adsorption phase followed by a gradual approach to equilibrium over tens of minutes to approximately one hour, typically characterized by pseudo-second-order kinetics. Similar time-dependent decolorization profiles (at 497 nm) and brief equilibrium durations have been recorded for CR adsorption systems, encompassing waste-shell-based adsorbents and associated inorganic/biogenic materials.^41,42^

**Fig. 5:**
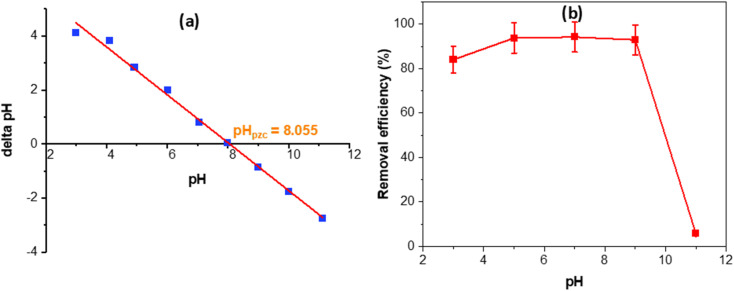
pH_pzc_ (a) and Congo red removal efficiency of MOS *vs.* pH (b).

The pH-drift curve (Fig. 5a) indicates a point of zero charge (pH_pzc_) of approximately 8.055, implying that the MOS surface is positively charged below this value and becomes negatively charged at higher pH. This surface charge transition governs the pH-dependent adsorption behavior shown in Fig. 5b. Under acidic to near-neutral conditions (pH 3–8), the positively charged MOS surface electrostatically attracts the anionic sulfonate groups of Congo red, resulting in high removal efficiencies of about 85–95%. The abundant –OH and phosphate groups identified by FTIR and XRD can also undergo protonation, enabling hydrogen bonding and ion exchange that further enhance dye affinity. As pH approaches the pH_pzc_ (≈8–9), charge neutralization reduces the driving force for adsorption, leading to a moderate decline in removal. At pH ≥ 9–10, the surface becomes negatively charged, and electrostatic repulsion, along with competitive adsorption by OH^−^ ions, suppresses dye uptake to below 10% at pH 11.

This pronounced pH dependence confirms that electrostatic interactions dominate the adsorption mechanism, while the presence of phosphate groups contributes to binding strength and stability. Importantly, because most industrial effluents exhibit near-neutral pH (6–8), the basic pH_pzc_ of MOS ensures that the adsorbent remains positively charged and highly effective under real wastewater conditions. This finding demonstrates the practical relevance and functional advantage of MOS over conventional high-surface-area materials such as activated carbon or MOFs, which often require extensive pH control or synthesis complexity. Hence, the MOS material combines low cost, simple preparation, and strong anion affinity, underscoring its promise as a sustainable and efficient adsorbent for dye-contaminated waters.

**Fig. 6:**
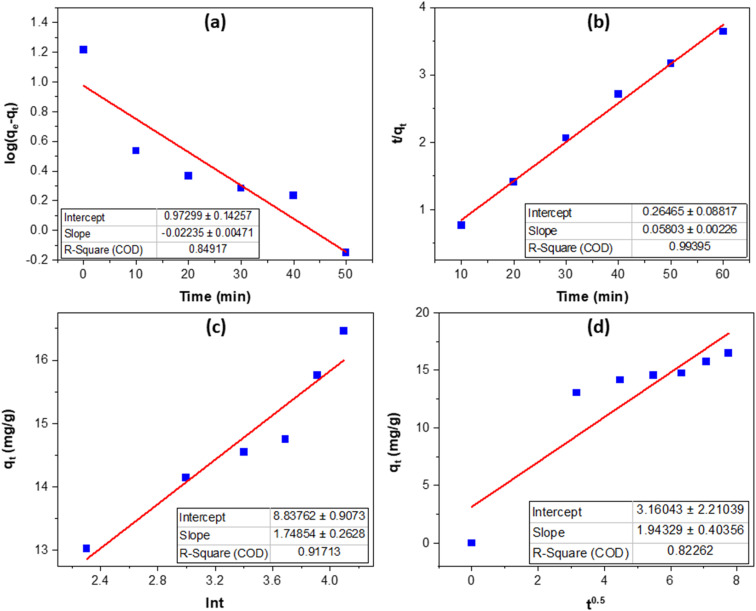
Kinetic models of Congo red adsorption on MOS: Psuedo-first order (a); Psuedo-second order (b); Elovich (c); and intrapartical diffusion (d).

Kinetic fitting results (Fig. 6, Table 1) show that Congo red adsorption on MOS is best described by the pseudo-second-order (PSO) model (*R*^2^ = 0.9940), with an equilibrium capacity of 17.23 mg g^−1^. The pseudo-first-order (PFO) model (*R*^2^ = 0.8492) underestimates capacity, confirming that adsorption is not dominated by simple physisorption. The strong PSO correlation suggests that the rate is mainly controlled by surface interactions—chemisorption-type processes such as ion exchange and electrostatic attraction between anionic CR and protonated phosphate or hydroxyl groups on MOS. This agrees with FTIR and XRD evidence of reactive –OH and PO_4_ sites introduced by phosphoric acid modification.

**Table 1:** Parameter of Congo red adsorption kinetic models on modified oyster shell

Model	Parameter	Value
Psuedo-first order	*k*_1_ (min^−1^)	0.0515
	*q*_e_ (mg g^−1^)	9.3970
	*R* ^2^	0.8492
Psuedo-second order	*k*_2_ (g mg^−1^ min^−1^)	0.0127
	*q*_e_ (mg g^−1^)	17.2325
	*R* ^2^	0.9940
Elovich	*a* (mg g^−1^)	8.8376
	*b* (g mg^−1^)	1.7485
	*R* ^2^	0.9171
Intrapartical diffusion	*k*_i_ (mg g^−1^ min^−0.5^)	1.9433
	*C* (mg g^−1^)	3.1604
	*R* ^2^	0.8226

The Elovich model (*R*^2^ = 0.9171) supports a heterogeneous surface, with rapid adsorption on high-energy sites followed by slower saturation. Intraparticle diffusion analysis (*k*_i_ = 1.94 mg g^−1^ min^−0.5^, *C* = 3.16 mg g^−1^) shows a non-zero intercept, indicating that both film and pore diffusion contribute but neither dominates.

Overall, the hierarchy PSO > Elovich > PFO/IPD confirms that adsorption is primarily surface-driven. Despite its moderate surface area, MOS exhibits fast and efficient dye uptake due to its phosphate-rich, reactive surface—addressing concerns about adsorption efficiency and validating its kinetic behavior.

**Fig. 7:**
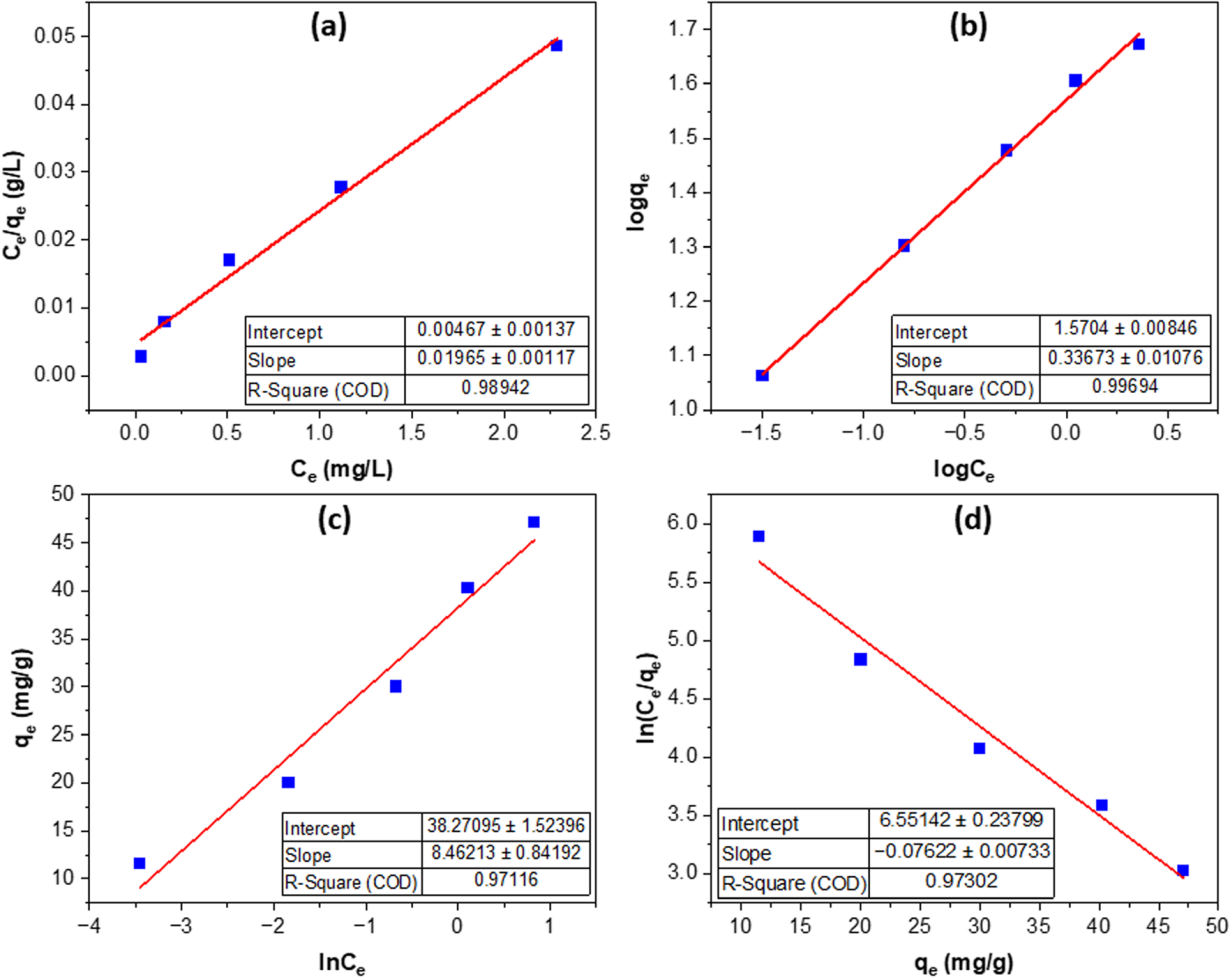
Isotherm models of Congo red adsorption on MOS: Langmuir (a), Freundlich (b), Temkin (c), and Elovich (d).

The equilibrium adsorption data of Congo red on MOS (Fig. 7, Table 2) fit both the Freundlich and Langmuir isotherm models well, with the Freundlich model exhibiting the best correlation (*R*^2^ = 0.9969). The Freundlich constants (*K*_F_ = 37.19, *n* = 2.97 > 1) indicate highly favorable adsorption on a heterogeneous surface possessing multiple high-energy binding sites. This observation is consistent with the surface roughness and phosphate-rich functional groups confirmed by SEM, FTIR, and XRD analyses.

**Table 2:** Parameter of isotherm models of Congo red adsorption on MOS

Models	Parameter	Value
Langmuir	*K*_L_ (L mg^−1^)	4.2077
	*q*_max_ (mg g^−1^)	50.89
	*R* ^2^	**0.9894**
Freundlich	*K*_F_ (mg g^−1^) (L mg^−1^)^1/*n*^	37.1877
	*N*	2.9697
	*R* ^2^	**0.9969**
Temkin	*K*_T_	92.076
	*b*_T_ (J mol^−1^)	8.4621
	*R* ^2^	0.9712
Elovich	*α* (mg g^−1^)	700.24
	*β* (g mg^−1^)	0.0762
	*R* ^2^	0.9730

The Langmuir model also shows a strong fit (*R*^2^ = 0.9894), giving a monolayer adsorption capacity of 50.89 mg g^−1^ and a high affinity constant (*K*_L_ = 4.21 L mg^−1^), reflecting strong and specific interactions between CR and the uniformly distributed active sites created during phosphoric acid modification. Collectively, the two models suggest that adsorption primarily occurs as monolayer coverage on an energetically heterogeneous surface, where electrostatic attraction and ion exchange are the dominant forces.

The Temkin model (*R*^2^ = 0.9712; *b*_T_ = 8.46 J mol^−1^) indicates a gradual decrease in adsorption energy with increasing surface coverage, while the Elovich model (*R*^2^ = 0.9730; *α* = 700.24 mg g^−1^; *β* = 0.0762 g mg^−1^) supports a chemisorption-type mechanism involving heterogeneous sites. The overall hierarchy (Freundlich ≳ Langmuir > Temkin ≈ Elovich) highlights the coexistence of monolayer and site-energy–distributed adsorption. These findings, together with the high *q*_max_ and strong affinity constants, confirm that MOS exhibits excellent adsorption efficiency due to its chemically active phosphate-modified surface—addressing reviewer concerns regarding biosorbent performance and mechanistic validity.

The maximum adsorption capacities summarized in Table 3 show that the modified oyster shell (MOS) achieves a *q*_max_ of 50.89 mg g^−1^, outperforming all conventional biosorbents listed. Low-cost materials such as the microwave rice husk clay hybrid (4.01 mg g^−1^), water hyacinth carbons (13.91–14.37 mg g^−1^), and marine-algae-derived sorbents including *Sargassum dentifolium* (28.24 mg g^−1^) and *Ulva fasciata* (30.95 mg g^−1^) exhibit relatively modest capacities, reflecting their limited density of active sites and predominantly physisorption-driven uptake. Even advanced iron oxide–carbon hybrids—homophase (17.95 mg g^−1^) and hetero-phased composites (45.84 mg g^−1^)—remain inferior to MOS despite their enhanced electrostatic affinity and surface hydroxyl groups.

**Table 3:** Maximum Congo red adsorption capacity of some adsorbents

Adsorbent	*q*_max_ (mg g^−1^)	References
Microwave rice husk clay hybrid (MRHCH)	4.008	41
Carbon from water hyacinth leaf	13.908	43
Carbon from water hyacinth stem	14.367	43
*Sargassum dentifolium*	28.24	44
Raw date pits	30.86	44
*Ulva fasciata*	30.95	44
Homophase iron oxide/carbon nanocomposite	17.95	45
Hetero-phased iron oxide/carbon	45.84	46
Imidazole-capped superparamagnetic α-Fe_2_O_3_	40.44	46
Modified oyster shell	50.89	This study
Cu–MOF	119.76	47
Co/Fe–MOF	530	48

More importantly, comparison with MOF-based adsorbents further contextualizes the performance of MOS. Representative MOFs such as Cu–MOF display capacities of approximately 119.76 mg g^−1^, while high-performance bimetallic systems like Co/Fe–MOF can reach extremely high *q*_max_ values (∼530 mg g^−1^). These values significantly exceed those of typical biosorbents and highlight the exceptional porosity and tunable chemistry of MOFs. However, such materials generally require complex synthesis routes, high-purity metallic precursors, and often involve organic linkers that limit scalability and economic feasibility for real wastewater treatment. Additionally, their regeneration stability and cost-per-cycle are typically less favorable compared to biosorbent-based systems.

Compared with both conventional biosorbents and engineered nanocomposites, MOS demonstrates a balanced and practically significant performance: although its *q*_max_ is lower than that of MOFs, it surpasses all other low-cost and biogenic materials examined, while being synthesized from an abundant waste resource through simple thermal–acid activation. The conversion of CaCO_3_ into Ca–phosphate phases introduces protonatable –OH and PO_4_ functional groups, enabling strong electrostatic attraction and ion-exchange interactions with the anionic sulfonate groups of Congo red. This surface-chemistry-driven enhancement—rather than merely high surface area—explains the superior capacity of MOS relative to other biosorbents and validates its adsorption mechanism as evidenced by FTIR, XRD, and pH_pzc_ analyses.

Therefore, MOS provides an effective and sustainable alternative to high-cost MOFs: it combines competitive adsorption capacity, rapid kinetics, broad pH applicability, and strong reusability with low production cost and environmentally friendly synthesis. These advantages reinforce the practical relevance of MOS for large-scale dye-laden wastewater treatment and directly address the reviewer's concern regarding the comparative performance of biosorbents relative to advanced MOF systems.

**Fig. 8:**
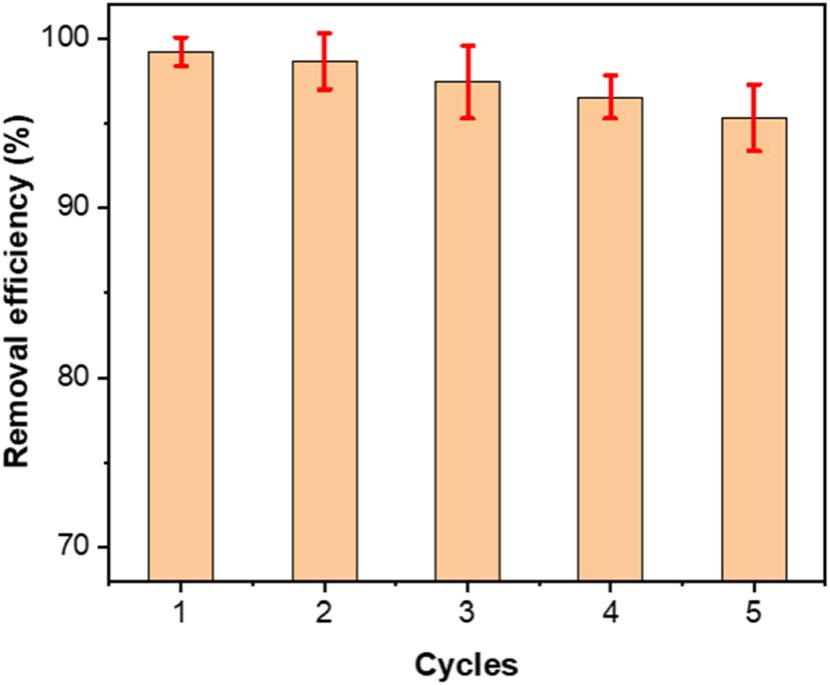
Reusability of MOS for Congo red adsorption.

As shown in Fig. 8, MOS maintains excellent reusability for Congo red removal over five consecutive adsorption–desorption cycles. The removal efficiency decreases only slightly from 99.21% in the first cycle to 95.32% in the fifth, corresponding to 96% retention of its initial performance. The narrow error bars indicate high reproducibility and consistent sorbent behavior. The minor decrease is attributed to incomplete desorption of strongly bound dye molecules, partial blocking of high-energy active sites by residual organics, and limited particle loss during handling, rather than any intrinsic structural deterioration.

This stable performance reflects the robustness of the phosphate-modified Ca–phosphate surface, whose protonated –OH and PO_4_ groups remain chemically intact during alkaline regeneration. Such chemical stability under repeated regeneration distinguishes MOS from many low-cost biosorbents, which typically lose activity after several cycles due to carbonate dissolution or surface deactivation. The combination of high retention, structural resilience, and simple regeneration validates MOS as a durable and sustainable adsorbent, suitable for cyclic treatment of dye-contaminated wastewater and scalable water purification systems.

## Conclusion

This study presents a scalable route to transform waste oyster shells into an efficient adsorbent for removing Congo red from water. Thermal calcination followed by H_3_PO_4_ activation converts the biogenic CaCO_3_ matrix into Ca-phosphate–rich surfaces verified by XRD/FTIR, yielding fractured morphologies with abundant –OH/PO_4_ sites. Despite lower BET area, the modified oyster shell (MOS) shows rapid uptake (>80–90% decolorization in 10 min; nearly complete by 60 min) and broad pH tolerance, optimal below pH_pzc_ = 8.06. Kinetics follow a pseudo-second-order model (*R*_2_ = 0.994; *k*_2_ = 0.0127 g mg^−1^ min^−1^). Equilibrium fits both Freundlich (*R*_2_ = 0.997; *K*_F_ = 37.19; *n* = 2.97) and Langmuir isotherms, giving *q*_max_ = 50.89 mg g^−1^ and a high affinity *K*_L_ = 4.21 L mg^−1^. MOS is durable and regenerable, retaining ∼95% removal after five adsorption–desorption cycles. Thus, thermal + acid activation yields a low-cost, reusable sorbent with fast kinetics, high affinity, and robustness, advancing circular-economy goals. Given benign precursors and straightforward processing, MOS is a practical candidate for dye-laden wastewater treatment. In addition, the mesopore size of MOS (>10 nm) is significantly larger than the molecular dimensions of Congo red, ensuring full accessibility of active sites without steric hindrance. The competitive *q*_max_ of MOS compared with several engineered porous materials, including iron oxide–carbon composites and certain MOFs, further confirms that phosphate-driven surface chemistry plays a more decisive role than extreme porosity. These findings highlight that MOS is not only an efficient biosorbent but also a scalable, low-cost, and environmentally compatible alternative for dye removal in practical wastewater treatment. Future work: validate fixed-/continuous-flow, quantify co-ion and NOM effects in real effluents, optimize greener regenerates, assess long-term stability, and perform techno-economic and life-cycle analyses to de-risk scale-up and real operating conditions worldwide.

## Conflicts of interest

There are no conflicts to declare.

## Data Availability

Data for this article, including SEM, XRD, BET, FTIR, …, and adsorption performance are available at Open Science Framework at https://osf.io/yht5a/overview.
